# Menstrual Cycle Characteristics and Injury History in Adult Amateur Female Football Players: A Cross-Sectional Study Using Selected LEAF-Q Items

**DOI:** 10.3390/healthcare14060773

**Published:** 2026-03-19

**Authors:** Joanna Witkoś, Joanna Kubik, Magdalena Hartman-Petrycka

**Affiliations:** 1 Department of Physical Medicine, Faculty of Health Sciences in Katowice, Medical University of Silesia, 40-055 Katowice, Poland; 2 Department of Nursing, State University of Applied Sciences in Krosno, Rynek Street 1, 38-400 Krosno, Poland; 3 Department of Basic Biomedical Science, Faculty of Pharmaceutical Sciences in Sosnowiec, Medical University of Silesia, 40-055 Katowice, Poland; mhartman@sum.edu.pl

**Keywords:** amateur women’s football players, menstrual health, amenorrhea, low energy availability, LEAF-Q

## Abstract

**Background/Objectives**: Increasing training demands in women’s football have heightened interest in female-specific health characteristics, including menstrual health. The aim of this study was to describe menstrual-cycle characteristics and injury history in adult amateur female football players using selected items of the Low Energy Availability in Females Questionnaire (LEAF-Q), with particular focus on prolonged absence of menstrual bleeding and training-associated menstrual changes. **Methods**: A cross-sectional survey was conducted in 118 adult amateur (non-elite) female football players (mean age 24.41 ± 4.50 years). Participants reported mean weekly training hours of 4.88 ± 2.45, consistent with amateur-level competitive and recreational participation. Selected items of the LEAF-Q were used, rather than the complete questionnaire; therefore, findings should be interpreted as descriptive indicators of menstrual health and injury history rather than a comprehensive LEA screening. **Results**: Most participants reported normal menstruation (95.76%), and menarche most commonly occurred between 12 and 14 years of age (92.37%). A history of ≥3 consecutive months without menstrual bleeding (clinically meaningful amenorrhea) was reported by 12.71% of players, while 4.24% reported such an episode at the time of the survey. Training-associated changes in menstrual bleeding were reported by 52.54% of participants, most commonly shorter and lighter bleeding; less frequently, cessation of bleeding (8.93%) or heavier and prolonged bleeding (1.79%) was reported. Injuries in the preceding 12 months were common, with 71.19% reporting one or two injuries and 28.81% reporting three or four injuries. **Conclusions**: Despite a high prevalence of self-reported regular menstrual cycles, a notable proportion of adult amateur female football players reported episodes of prolonged absence of menstrual bleeding and training-associated changes in bleeding characteristics. These findings highlight the variability of menstrual-cycle characteristics in the context of football training and support the inclusion of routine, confidential menstrual-health monitoring as part of broader athlete health management in women’s football. Football-related injuries were common over the preceding 12 months, reflecting the substantial musculoskeletal demands of the sport.

## 1. Introduction

In recent years, women’s football has undergone dynamic development, accompanied by an increase in both participation and training loads [1,2]. Contemporary analyses describe this period as transformative, characterised by progressive professionalisation and rising training and match-play demands across leagues worldwide [1,2]. Match play in women’s football is characterised by an intermittent high-intensity profile, in which technical and tactical actions are closely coupled with substantial mechanical and physiological loads. Although total distance covered during a match varies according to competitive level, age group, and measurement methodology, outfield players typically cover approximately 10 km per match [3,4]. Central midfielders generally accumulate the greatest total distance and highest volumes of high-intensity running, whereas central defenders tend to record the lowest values, underscoring the importance of interpreting external load within the context of positional role [3,4]. However, total running distance alone does not fully capture the key determinants of player load, as match outcomes are frequently influenced by high-intensity episodes, including sprinting efforts that constitute a meaningful proportion of match activity. The substantial match and training demands, together with the associated physical challenges, have intensified interest in health issues specific to female football players [5,6,7]. A growing body of evidence suggests that sex-specific physiological factors, including hormonal regulation, may play an important role in both adaptation to training and susceptibility to musculoskeletal injury. Nevertheless, the development of the sport has outpaced research conducted directly in female footballers, resulting in persistent knowledge gaps regarding optimal strategies to support athlete health and performance. Furthermore, increasingly congested competition schedules heighten the need for evidence-based training, medical, and nutritional approaches tailored to female physiology [7,8,9].

In athletic populations, prolonged mismatch between energy intake and exercise energy expenditure may lead to low energy availability (LEA) [10,11], which has been associated with disruptions in endocrine function and menstrual-cycle regulation. These phenomena are encompassed within the Female Athlete Triad framework, which includes energy deficiency, menstrual disturbances, and reduced bone mineral density; importantly, even the presence of a single component may adversely affect athlete health, impair recovery capacity, and co-occur with increased injury risk. Within the broader framework of Relative Energy Deficiency in Sport (REDs) [12], such disturbances are recognised as part of a wider spectrum of health consequences affecting multiple physiological systems. However, assessment of energy intake, energy expenditure, or energy availability was beyond the scope of the present study; therefore, these concepts are referenced solely to provide a contextual background for the interpretation of menstrual-cycle characteristics in women training football.

In female athletes, increasing attention has been directed toward sex-specific health considerations, including optimal hormonal function and reproductive health, often reflected in regular menstrual cycles. Menstrual-cycle function in athletes is regulated by the hypothalamic–pituitary–ovarian (HPO) axis, which is sensitive to physiological stressors, including training load and recovery demands [13]. Within athletic populations, menstrual-cycle disturbances represent one of the key health concerns affecting female athletes and are frequently overlooked or misinterpreted as a normal physiological consequence of intensive training. The most commonly reported sport-related menstrual disturbances include amenorrhea (absence of menstruation, e.g., ≥3 consecutive months without bleeding), oligomenorrhea (infrequent menstruation), cycle irregularity, and shortened or prolonged cycle length [14,15]. These disturbances may occur on a transient or chronic basis and are often associated with high training loads, low energy availability, and alterations in hormonal regulation. In contrast, menstrual symptoms include features such as dysmenorrhea, heavy menstrual bleeding, premenstrual mood changes, or fatigue, which may occur even in otherwise regular, ovulatory cycles.

Research conducted in women’s football indicates that menstrual-cycle irregularities and alterations in bleeding patterns are not uncommon and may exhibit considerable variability depending on the sporting context. Inter-individual differences in load tolerance and physiological responses to exercise further emphasise the importance of monitoring menstrual health as an integral component of comprehensive health assessment in female football players [13,14,15]. Evidence across different stages of the athletic career suggests that menstrual-cycle patterns and the nature of menstrual symptoms may vary according to age, developmental phase, and career trajectory. At the same time, available findings indicate that menstrual disturbances and associated symptom burden may constitute practical barriers both to sport participation and to open disclosure of concerns. Despite increasing interest in menstrual health within the context of athletic preparation, epidemiological data on the prevalence of menstrual disturbances in women’s football remain heterogeneous. This heterogeneity reflects differences in definitions, sampling strategies, inclusion criteria, and operationalisation of key concepts such as amenorrhea and oligomenorrhea. Even in elite cohorts, reported prevalence estimates vary depending on whether analyses focus on current status or on disturbances occurring at any point during the athlete’s career. Collectively, football-specific literature suggests that menstrual symptoms are common and often perceived as relevant to performance, while clinically meaningful menstrual-cycle disturbances occur in a non-negligible proportion of players across different career stages. Communication barriers may further limit early identification and appropriate management of such issues [13,14,15].

From a reproductive health and injury-prevention perspective, there is a need for screening and observational research that characterises menstrual patterns in female football players and identifies potential warning signals, such as episodes of prolonged absence of menstrual bleeding, reduced menstrual frequency, or training-associated changes in bleeding characteristics. Although the Low Energy Availability in Females Questionnaire (LEAF-Q) [16] has been widely used as a screening tool in athletic populations, most studies have applied the complete instrument and have focused primarily on elite athletes. Consequently, relatively little is known about menstrual-cycle characteristics and injury history in adult amateur female football players who are not using hormonal contraception. Therefore, the aim of the present study was to provide a descriptive overview of menstrual-cycle characteristics and injury history in adult amateur female football players using selected items of the LEAF-Q, with particular emphasis on cycle regularity, prolonged absence of menstrual bleeding (≥3 months), and training-associated changes in bleeding patterns, as self-reported by the participants.

## 2. Materials and Methods

### 2.1. Participants

A total of 118 adult amateur female football players were included in the study. The mean age of participants was 24.41 years (standard deviation [SD] 4.50). Mean height was 168.37 cm (SD 5.68), and mean body mass was 62.10 kg (SD 6.56). At their current height, participants retrospectively reported the highest and lowest body mass achieved during adulthood and throughout their football career. The mean highest self-reported body mass was 65.48 kg (SD 7.22), whereas the mean lowest self-reported body mass was 57.61 kg (SD 5.64). Body mass values were obtained via self-report and were not directly measured. No information was collected regarding the timing or duration of these body mass fluctuations. The mean body mass index (BMI) was 21.91 kg/m^2^ (SD 2.16). Players reported a mean weekly training volume of 4.88 h (SD 2.45).

Participants were recruited from amateur football clubs and teams located in two neighbouring provinces in southern Poland. Participation in the study was voluntary. Data collection was conducted within the same time frame for all participants, between January and February, corresponding to the transition from the off-season recovery period to the early preparatory phase, after which players entered the pre-season period. Typical training exposure consisted of regular team-based training sessions, and the reported weekly training volume reflected the amateur competitive context. Study information was disseminated to players with the assistance of coaching staff; however, given the sensitive nature of menstrual-health data collected in the study, coaches had no access to individual responses. To ensure full protection of participants’ privacy, the questionnaire was administered anonymously online, and no directly identifying information was collected. Data collection took place outside the training environment, and participants were encouraged to complete the survey privately and to provide accurate responses. No time limit was imposed, and respondents were free to withdraw from the study at any time without consequences. The dataset was stored on a password-protected drive accessible only to members of the research team. All procedures were designed in accordance with current good-practice recommendations for menstrual-health monitoring in football, with emphasis on confidentiality, respect, and minimisation of potential discomfort. After reviewing the study information, eligible individuals accessed the online survey link and completed the questionnaire independently in a non-supervised setting, most commonly at home. Incomplete or incorrectly completed questionnaires were removed from the database prior to analysis. Match exposure was not quantified, and years of playing experience and playing position were not recorded. Training exposure was based solely on self-reported weekly training hours.

Adult (≥18 years) amateur female football players who were actively training were eligible for inclusion in the study. Regular participation was defined as ongoing involvement in organised football training, comprising at least one training session per week. Exclusion criteria included current use of hormonal contraception, self-reported amenorrhea attributable to causes unrelated to sport participation (e.g., pregnancy, polycystic ovary syndrome, hysterectomy), and age < 18 years.

### 2.2. Questionnaire

In the present study, a modified version of the LEAF-Q was used [16]. Specifically, the gastrointestinal domain was not administered, and only selected items from the menstrual-function and injury domains were included in the analysis. Thus, although the LEAF-Q was originally developed as a multidomain screening tool for low energy availability, a modified version of the instrument was applied in this study. As a result, calculation of the validated total LEAF-Q score and application of the standard ≥8 cut-off for increased low energy availability risk were not possible. Consequently, the present study should be interpreted as a descriptive assessment of menstrual-cycle characteristics and injury history based on selected LEAF-Q items, rather than as a comprehensive screening for LEA.

Because the questionnaire was completed independently at home via a distributed link, a proportion of submissions contained errors and had to be excluded from analysis. Missing or incomplete responses most frequently concerned the gastrointestinal domain. Therefore, to avoid discarding otherwise complete questionnaires containing valid data on menstrual-cycle characteristics and injury history, this domain was omitted for all participants, and analyses were based on selected LEAF-Q items addressing menstrual function and injuries. From a conceptual perspective, menstrual health and injury burden represent clinically relevant aspects in women’s football and can be meaningfully analysed independently of a full LEA screening framework. It should be emphasised that the selected items were not treated as validated subscales but rather as individual indicators used exclusively for descriptive purposes.

In the present study, the total LEAF-Q score was not used to classify LEA risk; instead, selected items were analysed descriptively in line with the study aim. In the present study, menstrual-cycle dysfunctions were defined as disturbances in menstrual cyclicity and/or cycle frequency and were operationalised using LEAF-Q menstrual-function items as follows: lack of cycle regularity (item C2, defined in the questionnaire as cycles not occurring every 28–34 days), reduced annual cycle frequency (item C5), and prolonged absence of menstrual bleeding (item D; ≥3 consecutive months without menstruation, excluding pregnancy). In contrast, bleeding characteristics were defined as features of menstruation that may occur with or without altered cyclicity and included bleeding duration (item C3), self-reported history of heavy menstrual bleeding (item C4), and perceived training-related changes in bleeding pattern (items E–E1; e.g., reduced bleeding volume, fewer bleeding days, or cessation of menstruation with increased training intensity, frequency, or duration). Timing of the last menstrual period was also recorded (item C1 or C6, depending on whether participants reported “normal menstruation”). Importantly, this section of the LEAF-Q does not assess symptom burden such as pain severity or mood-related or premenstrual symptoms. Accordingly, the present study focused on menstrual-cycle characteristics and bleeding-pattern indicators rather than comprehensive menstrual symptomatology.

In the present study, injury characteristics were operationalised using selected items from the “Injuries” domain of the LEAF-Q. Injuries were defined as musculoskeletal complaints sustained during football training or match play that resulted in missing at least one training session or competition. Injury frequency was assessed based on item A, which addressed the number of training or match absences due to injury within the previous 12 months (categorical responses: none, 1–2 times, 3–4 times, ≥5 times). The temporal severity of injury was described using item A1, referring to the total number of days of absence during the preceding year (1–7 days, 8–14 days, 15–21 days, ≥22 days). Injury type was reported descriptively (item A2), allowing qualitative identification of the most common anatomical locations and injury types (e.g., ankle sprains, knee injuries, muscle injuries, fractures). As with menstrual variables, injury-related items were analysed as individual descriptive indicators. No standardised epidemiological classification (e.g., Fédération Internationale de Football Association (FIFA) or Union of European Football Associations (UEFA) consensus definitions) was applied, and data were retrospective and self-reported, covering the 12 months preceding the survey [17,18]. Injuries were not verified through medical records, and no formal injury surveillance system was implemented. Accordingly, the findings should be interpreted as descriptive indicators of injury burden within the sample, rather than as epidemiologically validated incidence rates adjusted for training or match exposure.

In the present study, injury data are presented as descriptive, contextual information reflecting the musculoskeletal demands of football, rather than as analytical outcomes intended to examine associations with menstrual-cycle characteristics.

### 2.3. Statistical Analyses

Descriptive statistics were used to summarise the data (mean ± standard deviation [SD] for continuous variables and n [%] for categorical variables). Comparative analyses between players reporting a normal menstrual cycle and those reporting menstrual-cycle disturbances were not performed because the menstrual-cycle disturbance group was very small (n = 5), resulting in expected cell counts of <5 in contingency tables. Under these conditions, chi-square testing was not appropriate, and inferential comparisons would have had limited reliability and statistical power [19,20].

## 3. Results

### 3.1. Menstrual Cycle Characteristics

Among the 118 surveyed female football players, menarche occurred spontaneously in 95.76% (95% CI: 92.0–99.6%) of participants. Menarche most commonly occurred between 12 and 14 years of age (92.37%; 95% CI: 87.6–97.2%), whereas 7.63% reported menarche at ≥15 years of age; these respondents indicated that menstrual bleeding commenced only following hormonal treatment. Most players (n = 113; 95.76%; 95% CI: 92.0–99.6%) reported no menstrual cycle-related problems, while five participants (4.24%; 95% CI: 0.6–7.9%) declared menstrual irregularities. Overall, 95.76% reported that their most recent menstrual period had occurred within the preceding 0–4 weeks. All athletes reporting irregular cycles stated that their last menstrual period had occurred 3–4 months prior to survey completion. The menstrual cycle was most commonly described as regular, with a typical cycle length of 28–34 days. Menstrual bleeding duration was reported as 3–9 days or longer by all participants, and none reported heavy menstrual bleeding. With respect to menstrual frequency, 80.51% of participants reported 12 or more cycles in the previous year, 15.25% reported 9–11 cycles, and 4.24% reported 6–8 cycles.

### 3.2. Menstrual Cycle Disturbances

The majority of athletes (87.29%; 95% CI: 81.27–93.31%) denied any episode of amenorrhea lasting longer than three months (excluding pregnancy). A history of prolonged absence of menstrual bleeding (≥3 consecutive months) was reported by 12.71% (95% CI: 6.7–18.7%) of participants. Among these, 8.47% indicated that such an episode had occurred prior to the study, whereas 4.24% (95% CI: 0.6–7.9%) reported experiencing prolonged absence of menstrual bleeding at the time of the survey.

### 3.3. Training-Related Menstrual Changes

Although the majority of participants self-reported “normal menstruation,” a notable proportion simultaneously reported a history of prolonged absence of menstrual bleeding and/or training-related menstrual changes, reflecting differences in how menstrual health was captured across questionnaire items. A total of 47.46% (95% Cl: 38.45–56.47%) of the participants reported perceived menstrual-cycle changes during periods of increased training intensity, frequency, or duration. Among those reporting changes, 89.29% described shorter and lighter menstrual bleeding, 1.79% reported heavier and longer bleeding, and 8.93% reported cessation of menstruation.

### 3.4. Injury History

In the preceding 12 months, one or two injuries were reported by 28.81% (95% CI: 20.6–37.0%) of players. The most frequently reported injuries included ankle sprains, knee injuries (including ligament ruptures), muscle strains or overuse-related muscle injuries, as well as dislocations and fractures. Injury-related absence from training was reported as lasting 1–7 days in 11.02% of players, 8–14 days in 3.39%, 15–21 days in 5.08%, and ≥22 days in 9.32%. Regarding missed training sessions or competitions due to injury, 12.71% reported one or two episodes, 14.41% reported three or four episodes, and 0.85% reported five or more episodes; the latter corresponded to a participant who also reported general health problems. The remaining athletes reported no injury-related absences from training or competition during the previous year. Descriptive characteristics stratified by menstrual-cycle status and by weekly training volume are presented in Table 1 and Table 2.

## 4. Discussion

Intensive physical activity associated with sport may exert multidimensional effects on women’s health, extending beyond musculoskeletal adaptations to encompass reproductive function and other sex-specific health domains. Consequently, there is growing interest in health-related issues affecting female athletes that may influence daily functioning, continuity of the training process, and overall quality of life, yet remain insufficiently recognised and monitored due to communication barriers and persistent taboos within the sporting environment. Across the scientific literature on female footballers, reported prevalence estimates of menstrual-cycle disturbances vary considerably depending on the definitions applied, players’ age and competitive level, and the assessment methodology used, including the mode of data collection. Such heterogeneity in definitions and classification represents a well-recognised limitation of menstrual-health research in football and athletic populations more broadly and should be taken into account when interpreting comparisons between studies [13,14,15,21,22,23,24,25,26,27].

In the present study, menstrual health and injury history were characterised in adult amateur female football players. Although the majority of respondents reported regular menstrual cycles, a subset indicated abnormalities in bleeding patterns, including episodes of prolonged absence of menstrual bleeding. More than half of the participants reported changes in menstrual-cycle characteristics during periods of increased training load, most commonly shorter and lighter bleeding. From a physiological perspective, shorter and lighter bleeding patterns have been described in the literature as potentially compatible with alterations in hypothalamic–pituitary–ovarian axis regulation under conditions of cumulative physiological stress. However, the cross-sectional design of the present study and reliance on self-reported data preclude inference regarding underlying endocrine mechanisms, as no objective hormonal assessments or confirmation of ovulation were performed. Therefore, the findings should be interpreted as reflecting subjective perceptions of menstrual-cycle variability rather than evidence of physiologically confirmed training-induced menstrual dysfunction.

The pattern observed in the present study, characterised by a relatively low prevalence of current menstrual-cycle disturbances alongside a noticeable frequency of reported training-associated changes in bleeding characteristics, is consistent with observations suggesting that menstrual disturbances in sport may be intermittent, time-varying, and responsive to load-related stressors. The discrepancy between the high proportion of players declaring “no problems” with their cycle and the concurrent reporting of abnormalities further indicates that broad, single-item questions regarding menstrual problems may underestimate the true prevalence of menstrual-cycle disturbances. Moreover, self-reported “normal menstruation” may lead to misclassification of menstrual-health status. In addition to recall bias inherent in retrospective data, athletes may lack awareness of subclinical menstrual abnormalities, such as anovulatory cycles or luteal phase defects, which cannot be identified without hormonal assessment or confirmation of ovulation. Consequently, the high proportion of participants reporting regular menstrual cycles does not exclude the presence of subtle endocrine alterations, which were not assessed in the present study.

Participants reported a mean weekly training volume of approximately 5 h. This level of training exposure would not typically be considered high in comparison with the demands characteristic of elite women’s football, therefore, the present findings should not be directly equated with or compared to data derived from professional cohorts. Nevertheless, the limited number of studies conducted in adult amateur female football players justified contextualising our descriptive characterisation of menstrual-cycle features, obtained in a cohort with moderate, self-reported training exposure, within the existing literature. This approach allows the present observations to be situated within the broader framework of menstrual-health research in women’s football, while acknowledging differences in competitive level and training demands. Compared with football-specific data, the prevalence of current prolonged absence of menstrual bleeding in our sample (4.24%), although a larger proportion reported having experienced such episodes in the past, underscoring the intermittent and time-varying nature of menstrual disturbances in sport, was similar in magnitude, albeit slightly higher than the 2% prevalence of secondary amenorrhea reported by Brown et al. [14] among elite footballers not using hormonal contraception. In the same cohort, oligomenorrhea was reported by 19% of players. These differences likely reflect definitional heterogeneity (e.g., “≥3 months without bleeding” versus broader categorizations of menstrual dysfunction) and/or differences in temporal framing, as some studies capture lifetime or career-based prevalence, whereas the present study primarily reports current prevalence at the time of survey administration, which likely contributes to the observed discrepancies. Notably, none of the participants in our study reported heavy menstrual bleeding, in contrast to the findings of Brown et al. [14], where 11% of players reported such symptoms. Heavy menstrual bleeding is particularly susceptible to underestimation when not assessed using structured criteria, such as validated questionnaire items or proxy indicators (e.g., flooding requiring very frequent changes of sanitary products, passage of large clots, or recurrent leakage).

In the present cohort of female football players, menarche most commonly occurred between 12 and 14 years of age (92.37%), whereas 7.63% of participants reported onset of menarche after the age of 15. Notably, these individuals indicated that menstrual bleeding commenced only following hormonal treatment. In the study by Brown et al. [14], the proportion of players reporting menarche at ≥15 years of age was higher (26%). For comparison, Prather et al. [22] reported a mean age at menarche of 13 ± 1 years in female soccer players, while also suggesting a tendency toward later menarche in this population despite normal BMI. Ramagole et al. [23], in a cohort of professional players, observed a mean age at menarche of 13.5 years, an average cycle length of 26 days, and a mean bleeding duration of approximately five days. Between-study differences may reflect variation in participant characteristics, including age distribution, competitive level, pre-menarche training history, and access to healthcare. Nevertheless, delayed menarche, particularly when reported as requiring hormonal induction, remains clinically relevant and should be considered within the broader context of athlete health. Available evidence indicates that delayed menarche is observed in women’s football and may represent an aspect of reproductive maturation that warrants attention in health-monitoring practices across different stages of athletic development [13,14,15,21,22,23,24,25,26,27].

One of the most informative findings of the present study was the high proportion of players (52.54%) reporting changes in menstrual-cycle characteristics during periods of increased training load, including greater intensity, frequency, and duration of training sessions. Among those reporting changes, shorter and lighter menstrual bleeding predominated (89.29%), whereas complete cessation of bleeding was reported less frequently. This pattern is consistent with the observations of Ling et al. [7], who demonstrated a reduced frequency of menstruation with increasing levels of physical activity in a substantial proportion of current and former football players. Collectively, these findings suggest that, in a sizeable subgroup of athletes, menstrual-cycle characteristics appear responsive to variations in training load, manifesting as reduced bleeding frequency and/or transient absence of menstruation during periods of intensified training. In light of the existing literature, shorter and lighter bleeding or prolonged intervals between bleeding episodes have been described as potential manifestations of altered menstrual function in physically active women. However, it should be emphasised that the present data do not permit conclusions regarding underlying physiological mechanisms, as cross-sectional, self-reported information does not allow differentiation between anovulatory cycles, luteal phase defects, or functional hypothalamic amenorrhea. These considerations reinforce the importance of systematic assessment of menstrual health as an integral component of comprehensive athlete health surveillance, aimed at identifying irregularities and supporting the individual-level interpretation of health- and training-related data [13]. In this context, the UEFA consensus statement on menstrual-cycle monitoring in women’s football emphasises that tracking should primarily serve to detect irregularities and early warning signs, while acknowledging that associations between menstrual-cycle phase and performance parameters are variable and highly individualised [28].

The menstrual cycle and menstruation may also constitute an underdisclosed topic, particularly in environments characterised by limited menstrual-health education or where players perceive insufficient support from coaching and medical staff. Recent research in women’s football has described barriers to communicating menstrual concerns and the coping strategies adopted by players, suggesting that both symptom burden and willingness to disclose influence what is reported, and how comprehensively, in questionnaire-based studies [13,21]. Notably, previous studies have shown that more than half of players reported limited confidence in staff knowledge and reduced trust in coaches and other support personnel when discussing menstrual health, while fear of unintended disclosure of such information was identified as one of the most frequently reported external barriers to sport participation.

The injury-related findings of the present study were based on retrospective self-report and were not collected using a standardized prospective injury-surveillance protocol, which limits their direct comparability with epidemiological studies conducted in elite football settings. Nevertheless, the reported injury burden in this cohort was substantial, with all participants indicating at least one or two injuries during the preceding 12 months. The injury profile was largely typical for football, with a predominance of ankle and knee injuries, as well as muscle overuse-related problems. Injury counts were presented as absolute self-reported numbers and were not normalised to individual training or match exposure; therefore, these data should be interpreted as descriptive indicators of injury burden rather than as incidence rates.

In light of current evidence, menstrual-cycle disturbances are increasingly recognised as factors that may indirectly modulate injury risk through hormonally mediated effects on bone metabolism, ligamentous laxity, and tissue-repair processes. One of the most clinically relevant consequences of menstrual dysfunction is impaired bone health, which may increase susceptibility to stress fractures and other overuse injuries. Evidence indicates that female athletes with a history of amenorrhea experience musculoskeletal injuries more frequently; however, menstrual disturbances are not always identified at an early stage or adequately incorporated into preventive strategies. This underscores the importance of considering menstrual-cycle dysfunction as a potential health risk factor rather than merely an adaptation to high levels of physical activity. Epidemiological analyses of injuries in women’s sport, including elite women’s football, consistently report a high incidence of injuries, particularly affecting the lower extremities, such as the knee, ankle, and thigh musculature, with ligament sprains and muscle injuries being the most common types. Although such studies do not always directly account for players’ hormonal status, they identify anatomical structures that are particularly vulnerable to overload, as well as potential biomechanical and structural alterations associated with injury risk.

Although the REDs framework [12] and the broader sports medicine literature describe associations between menstrual dysfunction and increased injury susceptibility, the cross-sectional design of the present study does not permit causal inference or robust evaluation of relationships between menstrual-cycle characteristics and injury occurrence. Importantly, evidence regarding the association between menstrual-cycle phase and injury risk remains inconsistent, partly due to definitional variability and challenges in accurately classifying cycle phases [29,30,31,32,33,34,35,36]. Therefore, the injury-related findings of the present study should be interpreted as clinically relevant contextual information reflecting the substantial musculoskeletal demands of football, rather than as evidence that menstrual-cycle disturbances directly translate into increased injury risk. For example, Barlow et al. [29] demonstrated phase-dependent differences in injury risk in a prospective cohort of elite female footballers, highlighting the potential practical value of menstrual-cycle monitoring. However, such monitoring should be considered complementary to, rather than a substitute for, established athlete health-management strategies described in the literature, including systematic monitoring of training load, recovery, and overall health history.

The findings of the present study underscore the need to direct greater attention to the reproductive health of women training for football. In particular, the results suggest that disturbances in menstrual bleeding patterns may occur more frequently than would be inferred from general self-declarations alone, and that menstrual-cycle characteristics in many players appear responsive to increases in training load. These observations are consistent with current recommendations advocating the implementation of routine, confidential menstrual-cycle monitoring within football clubs and academies, including assessment of cycle regularity, episodes of ≥3 consecutive months without bleeding, and changes in bleeding characteristics. Importantly, such information should be interpreted in conjunction with data on training exposure and recovery. From a sports medicine and injury-prevention perspective, these considerations highlight the need for screening and observational research that characterises menstrual patterns in female footballers and identifies potential warning signals, such as prolonged absence of menstrual bleeding, reduced menstrual frequency, or training-associated changes in bleeding characteristics. Contemporary guidelines and implementation reports indicate that regular menstrual-cycle monitoring is most valuable for detecting irregularities and informing symptom management, provided that ethical standards, confidentiality, and cultural sensitivity are ensured [13,21,37,38].

In summary, engagement in structured sports training may exert multidimensional effects on women’s health, extending beyond musculoskeletal adaptations to encompass endocrine, reproductive, and psychosocial domains. Among the “sensitive” areas potentially influenced by training load are pelvic-floor function and urinary continence, which have been described in high-level female athletes as clinically relevant concerns that may adversely affect quality of life and, in some cases, limit full participation in training [39]. Collectively, these observations support the implementation of athlete-centred, confidential health-monitoring systems in women’s sport, aimed at enabling early identification of sensitive health issues and facilitating timely access to appropriate clinical support.

### 4.1. Future Directions for Research

Future research in women’s football should adopt prospective, multidimensional study designs that integrate menstrual-cycle monitoring with objective assessment of training load and energy availability. Comprehensive REDs screening approaches, including validated questionnaires administered in full (e.g., the full version of the LEAF-Q), hormonal measurements, ovulation confirmation, dietary intake assessment, and estimation of exercise energy expenditure, would allow more precise classification of menstrual dysfunction and LEA risk.

Integration of menstrual tracking with load-monitoring systems and injury surveillance protocols could further clarify temporal relationships between training exposure, menstrual variability, and musculoskeletal outcomes. Such approaches would improve causal inference and enhance the development of evidence-based, athlete-centred monitoring strategies in women’s football.

### 4.2. Limitations

In the present study, a modified version of the LEAF-Q was employed. Specifically, omission of the gastrointestinal domain precluded calculation of the validated total LEAF-Q score and application of the standard ≥8 cut-off for increased risk of low energy availability. Accordingly, the findings should be interpreted as a descriptive assessment of menstrual health and injury history based on selected LEAF-Q items, rather than as a comprehensive screening for LEA or REDs. Additional limitations include reliance on self-reported data, which may be subject to recall bias, social desirability bias, and potential underreporting, particularly with respect to sensitive health-related issues such as menstrual function and injury history over the preceding 12 months. Furthermore, the cross-sectional design does not permit causal inference or evaluation of temporal relationships between training load, menstrual-cycle characteristics, and injury occurrence. The relatively small number of participants reporting current menstrual-cycle disturbances further limited the feasibility of inferential statistical analyses.

Match exposure and competition-related load were not directly assessed, which restricts more detailed interpretation of injury burden in relation to training and match demands. Training exposure was evaluated at a general level based on self-reported weekly training hours; however, match exposure, fixture congestion, years of playing experience, and playing position were not recorded. This limits the ability to examine their potential influence on menstrual-cycle characteristics and injury history. The absence of detailed information on match exposure, playing experience, and seasonal congestion precluded stratified descriptive analyses. Collectively, these methodological constraints reflect the exploratory and descriptive nature of the study and should be taken into account when interpreting the findings within the broader REDs and menstrual-health literature.

## 5. Conclusions

In this cohort of adult amateur female football players, most participants reported regular menstrual cycles; however, a meaningful proportion disclosed a history of prolonged absence of menstrual bleeding, and more than half perceived training-related menstrual changes, most commonly shorter and lighter bleeding. These findings indicate that variability in menstrual-cycle characteristics is frequently reported in the context of training demands in women’s football, and that self-reported “normal menstruation” may coexist with cycle irregularities or transient alterations. This underscores the value of routine menstrual-health monitoring in women’s football. Football-related injuries were common over the preceding 12 months and are presented as contextual information reflecting the musculoskeletal demands inherent to the sport.

As a modified version of the LEAF-Q was used, the present findings should be interpreted as a descriptive characterisation of menstrual health and injury history based on selected questionnaire items, rather than as a screening assessment for LEA or REDs. Nevertheless, the results support the implementation of confidential, athlete-centred menstrual-health monitoring as part of comprehensive health surveillance in women’s football, particularly during periods of increased training load.

## Figures and Tables

**Table 1:** Descriptive characteristics stratified by menstrual-cycle status; mean ± SD.

Variable	Regular Menstrual Cycle (n = 103)	Menstrual-Cycle Disturbance * (n = 15)
Age (years)	24.33 ± 4.55	24.93 ± 4.22
Body mass index (kg/m^2^)	21.64 ± 2.23	21.76 ± 1.73
Weekly training volume (h/week)	4.94 ± 2.52	4.47 ± 1.96
** Number of injuries (previous 12 months)		
No injuries, n (%)	84 (81.55%)	9 (60.00%)
1–2 injuries, n (%)	28 (27.18%)	6 (40.00%)
Injury-related training absence		
1–7 days, n (%)	8 (7.77%)	5 (33.33%)
8–14 days, n (%)	4 (3.88%)	0
15–21 days, n (%)	6 (5.83%)	0
≥22 days, n (%)	10 (9.71%)	1 (6.67%)

* Menstrual-cycle disturbance was defined as a self-reported history of ≥3 consecutive months without menstrual bleeding (current or past). Injury data are presented as absolute counts and were not normalised to training or match exposure. Prolonged absence of menstrual bleeding was defined as ≥3 consecutive months without menstruation, consistent with commonly used definitions of amenorrhea in sports medicine and REDs literature [12]. Oligomenorrhea (e.g., cycles > 35 days or <9 cycles/year) was not formally classified, as the study relied on selected questionnaire items and was not designed to provide a clinical diagnosis. ** Injuries were self-reported and defined as musculoskeletal problems sustained during football participation that resulted in training or match absence. Data were collected retrospectively and were not verified using medical records.

**Table 2:** Descriptive characteristics stratified by weekly training volume (h/week); mean ± SD.

Variable	Weekly Training Volume 1.5–4 h/Week (n = 69)	Weekly Training Volume 4.5–7.5 h/Week (n = 33)	Weekly Training Volume 8–14 h/Week (n = 16)
Age (years)	25.46 ± 5.28	23.09 ± 2.37	23.33 ± 2.32
Body mass index (kg/m^2^)	22.13 ± 2.17	21.86 ± 2.26	21.94 ± 2.23
Number of menstrual cycle			
Regular n (%)	60 (86.96%)	28 (84.85%)	15 (93.75%)
* Disturbance n (%)	9 (13.04%)	5 (15.15%)	1 (6.25%)
Training-related menstrual changes n (%)	26 (37.68%)	21 (63.64%)	9 (56.25%)
** Number of injuries (previous 12 months)			
No injuries, n (%)	53 (76,81%)	19 (57.58%)	12 (75%)
1–2 injuries, n (%)	16 (23.19%)	14 (42.42%)	4 (25%)
Injury-related training absence			
1–7 days, n (%)	2 (2.90%)	11 (33.33%)	0 (0.00%)
8–14 days, n (%)	2 (2.90%)	1 (3.33%)	1 (6.25%)
15–21 days, n (%)	4 (5.80%)	1 (3.33%)	1 (6.25%)
≥22 days, n (%)	8 (11.59%)	1 (3.33%)	2 (12.50%)

* Menstrual-cycle disturbance was defined as a self-reported history of ≥3 consecutive months without menstrual bleeding (current or past). Injury data are presented as absolute counts and were not normalised to training or match exposure. Prolonged absence of menstrual bleeding was defined as ≥3 consecutive months without menstruation, consistent with commonly used definitions of amenorrhea in sports medicine and REDs literature [12]. Oligomenorrhea (e.g., cycles > 35 days or <9 cycles/year) was not formally classified, as the study relied on selected questionnaire items and was not designed to provide a clinical diagnosis. ** Injuries were self-reported and defined as musculoskeletal problems sustained during football participation that resulted in training or match absence. Data were collected retrospectively and were not verified using medical records.

## Data Availability

The datasets used and/or analysed during the current study are available from the corresponding author upon reasonable request.

## Abbreviations

The following abbreviations are used in this manuscript:

LEAF-Q	Low Energy Availability in Females Questionnaire
LEA	low energy availability
REDs	Relative Energy Deficiency in Sport
HPO axis	hypothalamic–pituitary–ovarian axis
UEFA	Union of European Football Associations
BMI	body mass index
SD	standard deviation
